# 2-(2-Fluoro­biphenyl-4-yl)-*N*′-(propan-2-yl­idene)propanohydrazide

**DOI:** 10.1107/S1600536810009049

**Published:** 2010-03-17

**Authors:** Saira Khanum, Muhammad Farman, Nasim Hasan Rama, Shahid Hameed, Peter G. Jones

**Affiliations:** a Department of Chemistry, Quaid-i-Azam University, Islamabad 45320, Pakistan; b Institut für Anorganische und Analytische Chemie, Technische Universität Braunschweig, Postfach 3329, 38023 Braunschweig, Germany

## Abstract

In the title compound, C_18_H_19_FN_2_O, the hydrazide side chain is approximately perpendicular to the central ring [dihedral angle = 76.80 (5)°]. The F atom is disordered over two positions with occupancies of 0.818 (2) and 0.182 (2). The packing consists of chains of mol­ecules parallel to the *a* axis, connected by a bifurcated N—H⋯(O,N) hydrogen bond and a weak C_phen­yl_—H⋯O hydrogen bond. The packing is extended to a layer structure parallel to the *ab* plane by a weak C_phen­yl_—H⋯F hydrogen bond.

## Related literature

For the biological activity of hydrazides, see: Kumar *et al.* (2009 ▶); Galal *et al.*(2009 ▶); Bordoloi *et al.* (2009 ▶). For their use as inter­mediates in the synthesis of heterocyclic compounds, see: Küçükgüzel *et al.* (2007 ▶); Navidpour *et al.* (2006 ▶); Stocks *et al.* (2004 ▶). For details of the preparation, see: Furniss *et al.* (1989 ▶).
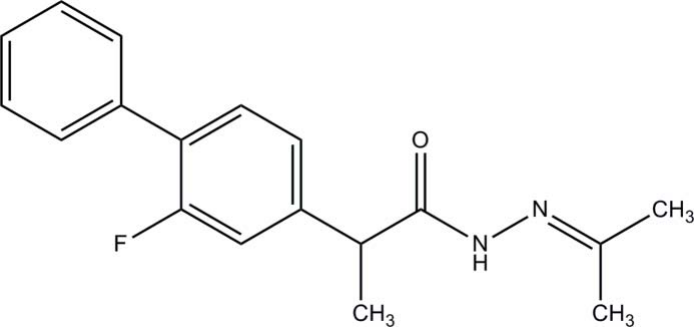


## Experimental

### 

#### Crystal data

C_18_H_19_FN_2_O
*M* *_r_* = 298.35
Orthorhombic, 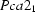

*a* = 7.5963 (3) Å
*b* = 7.3633 (3) Å
*c* = 27.7430 (11) Å
*V* = 1551.77 (11) Å^3^
*Z* = 4
Mo *K*α radiation
μ = 0.09 mm^−1^
*T* = 100 K
0.3 × 0.2 × 0.2 mm

#### Data collection

Oxford Diffraction Xcalibur E diffractometer
33827 measured reflections
2221 independent reflections
2019 reflections with *I* > 2σ(*I*)
*R*_int_ = 0.033

#### Refinement

*R*[*F* ^2^ > 2σ(*F* ^2^)] = 0.031
*wR*(*F* ^2^) = 0.076
*S* = 1.00
2221 reflections
210 parameters
2 restraints
H atoms treated by a mixture of independent and constrained refinement
Δρ_max_ = 0.26 e Å^−3^
Δρ_min_ = −0.17 e Å^−3^

### 

Data collection: *CrysAlis PRO* (Oxford Diffraction, 2009 ▶); cell refinement: *CrysAlis PRO*; data reduction: *CrysAlis PRO*; program(s) used to solve structure: *SHELXS97* (Sheldrick, 2008 ▶); program(s) used to refine structure: *SHELXL97* (Sheldrick, 2008 ▶); molecular graphics: *XP* (Sheldrick, 2008 ▶); software used to prepare material for publication: *SHELXL97*.

## Supplementary Material

Crystal structure: contains datablocks I, global. DOI: 10.1107/S1600536810009049/bt5206sup1.cif

Structure factors: contains datablocks I. DOI: 10.1107/S1600536810009049/bt5206Isup2.hkl

Additional supplementary materials:  crystallographic information; 3D view; checkCIF report

## Figures and Tables

**Table 1:** Hydrogen-bond geometry (Å, °)

*D*—H⋯*A*	*D*—H	H⋯*A*	*D*⋯*A*	*D*—H⋯*A*
N2—H01⋯O^i^	0.87 (3)	2.24 (3)	3.0633 (17)	158 (2)
N2—H01⋯N1^i^	0.87 (3)	2.45 (2)	3.0632 (17)	127.7 (19)
C6—H6⋯F^ii^	0.95	2.45	3.3537 (18)	159
C2—H2⋯O^i^	0.95	2.52	3.3816 (19)	150
C18—H18*A*⋯O^i^	0.98	2.29	3.251 (2)	166

Symmetry codes: (i) 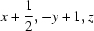; (ii) 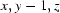.

**Fig. 1.**
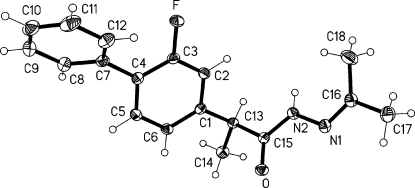
The molecule of the title compound. Ellipsoids correspond to 50% probability levels. The minor disorder component is omitted.

**Fig. 2.**
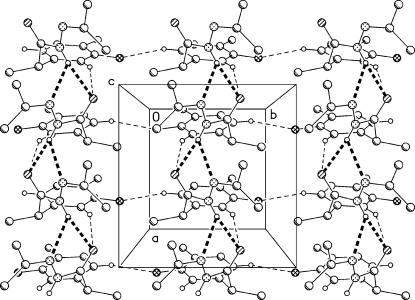
Packing diagram of the title compound viewed parallel to the z axis in the region z≈ 0. Classical (three-centre) H bonds are indicated by thick dashed lines and "weak" H bonds by thin dashed lines. For clarity, the ring C7–12 is represented only by the ipso C atom .

## supplementary crystallographic information

### Comment

Hydrazides represent one of the most biologically active class of compounds, possessing a wide spectrum of activities such as anti-microbial (Kumar *et al.*, 2009), anti-cancer (Galal *et al.*, 2009) and anti-genotoxic (Bordoloi *et al.*, 2009). They have been used as intermediates in the synthesis of a number of heterocyclic compounds such as oxadiazoles, triazoles and thiadiazoles (Küçükgüzel *et al.* 2007; Navidpour *et al.*, 2006; Stocks *et al.*, 2004). The title compound (I) was synthesized as an intermediate for onward conversion to 1,2,4-triazoles and 1,3,4-thiadiazoles and in order to explore their anti-bacterial, urease inhibition and anti-fungal activities.

The molecule of (I) is shown in Fig. 1. Molecular dimensions such as the bond lengths C16═N1 1.280 (2) or N1—N2 1.3896 (17) Å may be regarded as normal. The central ring C1–6 subtends interplanar angles of 44.25 (5)° with the ring C7–12 and 76.80 (5)° with the extended hydrazide moiety C13,15,16,17,N1,N2,O (r.m.s. deviation from latter plane 0.056 Å).

The N—H function of the hydrazide group acts as donor in a three-centre hydrogen bond to O and N1 of a molecule related by the *a* glide plane. The weak hydrogen bond C2—H2···O acts via the same operator, and these interactions lead to chains of molecules parallel to the *a* axis. The contact C6—H6···F via *b* axis translation connects the chains to form layers of molecules parallel to the *ab* plane (Fig. 2).

### Experimental

Methyl 4-ethoxybenzoate (0.02 moles) was dissolved in 40 ml methanol in a round-bottom flask fitted with a reflux condenser and a calcium chloride drying tube. Hydrazine hydrate (80%, 0.04 moles) was added slowly and the progress of the reaction was monitored by thin layer chromatography. After completion of the reaction, the contents were concentrated under reduced pressure (Furniss *et al.*, 1989). The resulting crude solid was filtered, washed with water and agitated with freshly distilled acetone for 1 h. The product was recrystallized from aqueous ethanol.

### Refinement

The NH hydrogen was refined freely. Methyl hydrogens were identified in difference syntheses, idealised and refined as rigid groups with C—H 0.98 Å and H—C—H angles 109.5°, allowed to rotate but not tip. Other hydrogens were placed in calculated positions and refined using a riding model with C—H_arom_ 0.95 and C—H_methine_ 1.00 Å; the hydrogen *U* values were fixed at 1.5 (methyl) or 1.2 × *U*(eq) of the parent atom.

The fluorine atom is disordered over the two sites at C3 and C5 with occupancies 0.818 (2),0.182 (2). The methyl hydrogens at C18 are treated as an idealised hexagon (disordered over two equally occupied sites) and the short contact to O may not be structurally significant.

In the absence of significant anomalous dispersion, the Friedel opposites were merged and the Flack parameter is thus meaningless.

### Crystal data

**Table:**

C_18_H_19_FN_2_O	*D*_x_ = 1.277 Mg m^−^^3^
*M**_r_* = 298.35	Melting point = 403–405 K
Orthorhombic, *P**c**a*2_1_	Mo *K*α radiation, λ = 0.71073 Å
*a* = 7.5963 (3) Å	Cell parameters from 14388 reflections
*b* = 7.3633 (3) Å	θ = 2.7–30.7°
*c* = 27.7430 (11) Å	µ = 0.09 mm^−^^1^
*V* = 1551.77 (11) Å^3^	*T* = 100 K
*Z* = 4	Block, colourless
*F*(000) = 632	0.3 × 0.2 × 0.2 mm

### Data collection

**Table:**

Oxford Diffraction Xcalibur E diffractometer	2019 reflections with *I* > 2σ(*I*)
Radiation source: Enhance (Mo) X-ray Source	*R*_int_ = 0.033
graphite	θ_max_ = 29.6°, θ_min_ = 2.8°
Detector resolution: 16.1419 pixels mm^-1^	*h* = −10→10
ω scan	*k* = −10→10
33827 measured reflections	*l* = −37→38
2221 independent reflections	

### Refinement

**Table:**

Refinement on *F*^2^	Primary atom site location: structure-invariant direct methods
Least-squares matrix: full	Secondary atom site location: difference Fourier map
*R*[*F*^2^ > 2σ(*F*^2^)] = 0.031	Hydrogen site location: inferred from neighbouring sites
*wR*(*F*^2^) = 0.076	H atoms treated by a mixture of independent and constrained refinement
*S* = 1.00	*w* = 1/[σ^2^(*F*_o_^2^) + (0.054*P*)^2^] where *P* = (*F*_o_^2^ + 2*F*_c_^2^)/3
2221 reflections	(Δ/σ)_max_ = 0.004
210 parameters	Δρ_max_ = 0.26 e Å^−^^3^
2 restraints	Δρ_min_ = −0.17 e Å^−^^3^

### Special details

**Table:**

Geometry. All esds (except the esd in the dihedral angle between two l.s. planes) are estimated using the full covariance matrix. The cell esds are taken into account individually in the estimation of esds in distances, angles and torsion angles; correlations between esds in cell parameters are only used when they are defined by crystal symmetry. An approximate (isotropic) treatment of cell esds is used for estimating esds involving l.s. planes.Least-squares planes (x,y,z in crystal coordinates) and deviations from them (* indicates atom used to define plane)6.3713 (0.0030) x + 4.0007 (0.0044) y - 1.0030 (0.0204) z = 5.8734 (0.0077)* -0.0031 (0.0011) C7 * 0.0023 (0.0012) C8 * 0.0002 (0.0014) C9 * -0.0018 (0.0013) C10 * 0.0009 (0.0012) C11 * 0.0016 (0.0012) C12Rms deviation of fitted atoms = 0.0019- 6.9258 (0.0030) x + 0.8494 (0.0036) y + 10.9376 (0.0231) z = 0.9826 (0.0143)Angle to previous plane (with approximate esd) = 44.25 ( 0.05 )* -0.0036 (0.0011) C1 * 0.0203 (0.0014) C2 * 0.0261 (0.0012) C3 * -0.0276 (0.0010) F_a * 0.0066 (0.0017) C4 * -0.0181 (0.0021) C5 * 0.0140 (0.0023) F'_b * -0.0177 (0.0014) C6Rms deviation of fitted atoms = 0.01863.4515 (0.0049) x - 4.4863 (0.0030) y + 18.0294 (0.0071) z = 11.4681 (0.0049)Angle to previous plane (with approximate esd) = 76.80 ( 0.05 )* -0.0272 (0.0009) C13 * 0.0140 (0.0012) C15 * -0.0802 (0.0013) C16 * -0.0112 (0.0011) C17 * 0.1063 (0.0013) N1 * 0.0372 (0.0012) N2 * -0.0387 (0.0006) ORms deviation of fitted atoms = 0.0557
Refinement. Refinement of *F*^2^ against ALL reflections. The weighted *R*-factor wR and goodness of fit *S* are based on *F*^2^, conventional *R*-factors *R* are based on *F*, with *F* set to zero for negative *F*^2^. The threshold expression of *F*^2^ > σ(*F*^2^) is used only for calculating *R*-factors(gt) etc. and is not relevant to the choice of reflections for refinement. *R*-factors based on *F*^2^ are statistically about twice as large as those based on *F*, and *R*- factors based on ALL data will be even larger.

### Fractional atomic coordinates and isotropic or equivalent isotropic displacement parameters (Å^2^)

**Table:**

	*x*	*y*	*z*	*U*_iso_*/*U*_eq_	Occ. (<1)
C1	0.72830 (18)	0.4169 (2)	0.51831 (5)	0.0147 (3)	
C2	0.7404 (2)	0.6049 (2)	0.51357 (5)	0.0174 (3)	
H2	0.7876	0.6764	0.5390	0.021*	
C3	0.68322 (19)	0.6863 (2)	0.47155 (5)	0.0176 (3)	
H3	0.6923	0.8146	0.4690	0.021*	0.182 (2)
F	0.70634 (17)	0.86695 (15)	0.46725 (4)	0.0264 (3)	0.818 (2)
C4	0.61277 (19)	0.5909 (2)	0.43257 (5)	0.0161 (3)	
C5	0.60263 (19)	0.4025 (2)	0.43852 (5)	0.0174 (3)	
H5	0.5562	0.3308	0.4131	0.021*	0.818 (2)
F'	0.5315 (7)	0.3049 (7)	0.40400 (17)	0.021*	0.182 (2)
C6	0.65842 (18)	0.31696 (19)	0.48053 (6)	0.0166 (3)	
H6	0.6485	0.1888	0.4834	0.020*	
C7	0.55385 (19)	0.6825 (2)	0.38780 (6)	0.0185 (3)	
C8	0.5944 (2)	0.6080 (2)	0.34264 (6)	0.0270 (3)	
H8	0.6626	0.5001	0.3406	0.032*	
C9	0.5349 (3)	0.6917 (3)	0.30082 (7)	0.0377 (5)	
H9	0.5624	0.6403	0.2703	0.045*	
C10	0.4364 (3)	0.8486 (3)	0.30319 (7)	0.0382 (5)	
H10	0.3962	0.9046	0.2744	0.046*	
C11	0.3960 (2)	0.9247 (3)	0.34731 (7)	0.0331 (4)	
H11	0.3284	1.0331	0.3489	0.040*	
C12	0.4548 (2)	0.8419 (2)	0.38960 (6)	0.0248 (3)	
H12	0.4270	0.8946	0.4199	0.030*	
C13	0.79126 (17)	0.33065 (19)	0.56537 (5)	0.0148 (3)	
H13	0.9016	0.3937	0.5757	0.018*	
C14	0.8295 (2)	0.1268 (2)	0.56215 (6)	0.0204 (3)	
H14A	0.7195	0.0609	0.5562	0.031*	
H14B	0.8814	0.0850	0.5925	0.031*	
H14C	0.9120	0.1039	0.5357	0.031*	
C15	0.64955 (18)	0.36582 (19)	0.60353 (5)	0.0145 (3)	
C16	0.5793 (2)	0.6916 (2)	0.69282 (6)	0.0204 (3)	
C17	0.4463 (2)	0.7287 (3)	0.73133 (7)	0.0292 (4)	
H17A	0.3519	0.6382	0.7295	0.044*	
H17B	0.3967	0.8503	0.7267	0.044*	
H17C	0.5031	0.7218	0.7630	0.044*	
C18	0.7172 (3)	0.8334 (3)	0.68449 (8)	0.0398 (5)	
H18A	0.7886	0.7998	0.6565	0.060*	0.50
H18B	0.7928	0.8426	0.7130	0.060*	0.50
H18C	0.6604	0.9508	0.6786	0.060*	0.50
H18D	0.7059	0.9290	0.7089	0.060*	0.50
H18E	0.7017	0.8862	0.6523	0.060*	0.50
H18F	0.8341	0.7779	0.6868	0.060*	0.50
O	0.51235 (13)	0.27749 (14)	0.60489 (4)	0.0192 (2)	
N1	0.56165 (16)	0.54268 (18)	0.66949 (5)	0.0185 (3)	
N2	0.68360 (16)	0.50682 (18)	0.63339 (5)	0.0169 (3)	
H01	0.785 (3)	0.562 (3)	0.6336 (8)	0.036 (6)*	

### Atomic displacement parameters (Å^2^)

**Table:**

	*U*^11^	*U*^22^	*U*^33^	*U*^12^	*U*^13^	*U*^23^
C1	0.0117 (6)	0.0169 (7)	0.0155 (7)	0.0008 (5)	0.0022 (5)	0.0009 (5)
C2	0.0180 (6)	0.0160 (7)	0.0182 (7)	−0.0017 (5)	0.0010 (5)	−0.0020 (5)
C3	0.0208 (6)	0.0132 (6)	0.0188 (7)	−0.0002 (5)	0.0030 (6)	0.0013 (6)
F	0.0436 (7)	0.0107 (5)	0.0249 (6)	−0.0022 (5)	−0.0030 (5)	0.0015 (4)
C4	0.0143 (6)	0.0174 (7)	0.0167 (7)	0.0012 (5)	0.0024 (5)	0.0000 (5)
C5	0.0167 (6)	0.0175 (7)	0.0180 (7)	−0.0011 (5)	−0.0003 (5)	−0.0024 (6)
C6	0.0173 (6)	0.0130 (6)	0.0195 (7)	0.0004 (5)	0.0012 (5)	−0.0010 (5)
C7	0.0172 (6)	0.0192 (7)	0.0192 (7)	−0.0033 (5)	−0.0012 (5)	0.0038 (6)
C8	0.0348 (9)	0.0253 (8)	0.0210 (8)	−0.0040 (7)	0.0003 (7)	0.0008 (7)
C9	0.0572 (12)	0.0355 (11)	0.0204 (8)	−0.0150 (9)	−0.0073 (8)	0.0029 (8)
C10	0.0443 (11)	0.0380 (10)	0.0324 (10)	−0.0160 (9)	−0.0173 (9)	0.0178 (9)
C11	0.0237 (8)	0.0333 (9)	0.0424 (11)	−0.0010 (7)	−0.0048 (8)	0.0176 (9)
C12	0.0212 (7)	0.0260 (8)	0.0273 (8)	0.0019 (6)	0.0020 (6)	0.0065 (7)
C13	0.0122 (6)	0.0156 (6)	0.0168 (7)	0.0001 (5)	0.0000 (5)	−0.0002 (5)
C14	0.0212 (7)	0.0167 (7)	0.0231 (8)	0.0042 (5)	0.0008 (6)	0.0007 (6)
C15	0.0139 (6)	0.0148 (6)	0.0147 (6)	0.0023 (5)	−0.0020 (5)	0.0019 (5)
C16	0.0188 (7)	0.0219 (7)	0.0206 (8)	0.0015 (6)	0.0003 (6)	−0.0009 (6)
C17	0.0313 (9)	0.0259 (8)	0.0303 (9)	0.0017 (7)	0.0109 (7)	−0.0046 (7)
C18	0.0399 (11)	0.0302 (9)	0.0492 (12)	−0.0126 (8)	0.0197 (9)	−0.0177 (9)
O	0.0148 (5)	0.0201 (5)	0.0226 (5)	−0.0028 (4)	0.0010 (4)	−0.0004 (5)
N1	0.0138 (5)	0.0228 (6)	0.0190 (6)	0.0019 (5)	0.0016 (5)	−0.0025 (5)
N2	0.0111 (5)	0.0207 (6)	0.0190 (6)	−0.0006 (5)	0.0004 (5)	−0.0036 (5)

### Geometric parameters (Å, °)

**Table:**

C1—C6	1.386 (2)	C2—H2	0.9500
C1—C2	1.394 (2)	C3—H3	0.9500
C1—C13	1.529 (2)	C5—H5	0.9500
C2—C3	1.381 (2)	C6—H6	0.9500
C3—F	1.3472 (18)	C8—H8	0.9500
C3—C4	1.396 (2)	C9—H9	0.9500
C4—C5	1.399 (2)	C10—H10	0.9500
C4—C7	1.482 (2)	C11—H11	0.9500
C5—F'	1.314 (5)	C12—H12	0.9500
C5—C6	1.391 (2)	C13—H13	1.0000
C7—C12	1.395 (2)	C14—H14A	0.9800
C7—C8	1.402 (2)	C14—H14B	0.9800
C8—C9	1.389 (3)	C14—H14C	0.9800
C9—C10	1.378 (3)	C17—H17A	0.9800
C10—C11	1.381 (3)	C17—H17B	0.9800
C11—C12	1.395 (2)	C17—H17C	0.9800
C13—C14	1.532 (2)	C18—H18A	0.9800
C13—C15	1.532 (2)	C18—H18B	0.9800
C15—O	1.2291 (17)	C18—H18C	0.9800
C15—N2	1.3530 (19)	C18—H18D	0.9800
C16—N1	1.280 (2)	C18—H18E	0.9800
C16—C17	1.495 (2)	C18—H18F	0.9800
C16—C18	1.497 (2)	N2—H01	0.87 (3)
N1—N2	1.3896 (17)		
			
C6—C1—C2	118.77 (13)	C9—C10—H10	119.9
C6—C1—C13	123.03 (13)	C11—C10—H10	119.9
C2—C1—C13	118.19 (12)	C10—C11—H11	120.1
C3—C2—C1	119.32 (13)	C12—C11—H11	120.1
F—C3—C2	117.53 (14)	C7—C12—H12	119.7
F—C3—C4	118.55 (14)	C11—C12—H12	119.7
C2—C3—C4	123.81 (14)	C1—C13—H13	108.2
C3—C4—C5	115.37 (13)	C14—C13—H13	108.2
C3—C4—C7	122.42 (13)	C15—C13—H13	108.2
C5—C4—C7	122.22 (13)	C13—C14—H14A	109.5
F'—C5—C6	119.2 (3)	C13—C14—H14B	109.5
F'—C5—C4	118.6 (3)	H14A—C14—H14B	109.5
C6—C5—C4	122.08 (13)	C13—C14—H14C	109.5
C1—C6—C5	120.65 (13)	H14A—C14—H14C	109.5
C12—C7—C8	118.66 (14)	H14B—C14—H14C	109.5
C12—C7—C4	121.03 (14)	C16—C17—H17A	109.5
C8—C7—C4	120.30 (14)	C16—C17—H17B	109.5
C9—C8—C7	120.09 (17)	H17A—C17—H17B	109.5
C10—C9—C8	120.58 (18)	C16—C17—H17C	109.5
C9—C10—C11	120.20 (17)	H17A—C17—H17C	109.5
C10—C11—C12	119.82 (17)	H17B—C17—H17C	109.5
C7—C12—C11	120.65 (16)	C16—C18—H18A	109.5
C1—C13—C14	114.63 (12)	C16—C18—H18B	109.5
C1—C13—C15	107.47 (11)	H18A—C18—H18B	109.5
C14—C13—C15	109.83 (12)	C16—C18—H18C	109.5
O—C15—N2	123.32 (14)	H18A—C18—H18C	109.5
O—C15—C13	121.84 (13)	H18B—C18—H18C	109.5
N2—C15—C13	114.74 (12)	C16—C18—H18D	109.5
N1—C16—C17	116.55 (14)	H18A—C18—H18D	141.1
N1—C16—C18	126.34 (15)	H18B—C18—H18D	56.3
C17—C16—C18	117.10 (15)	H18C—C18—H18D	56.3
C16—N1—N2	117.22 (13)	C16—C18—H18E	109.5
C15—N2—N1	117.36 (12)	H18A—C18—H18E	56.3
C3—C2—H2	120.3	H18B—C18—H18E	141.1
C1—C2—H2	120.3	H18C—C18—H18E	56.3
C2—C3—H3	118.1	H18D—C18—H18E	109.5
C4—C3—H3	118.1	C16—C18—H18F	109.5
C6—C5—H5	119.0	H18A—C18—H18F	56.3
C4—C5—H5	119.0	H18B—C18—H18F	56.3
C1—C6—H6	119.7	H18C—C18—H18F	141.1
C5—C6—H6	119.7	H18D—C18—H18F	109.5
C9—C8—H8	120.0	H18E—C18—H18F	109.5
C7—C8—H8	120.0	C15—N2—H01	122.2 (15)
C10—C9—H9	119.7	N1—N2—H01	119.6 (15)
C8—C9—H9	119.7		
			
C6—C1—C2—C3	−0.1 (2)	C4—C7—C8—C9	178.30 (16)
C13—C1—C2—C3	−179.52 (13)	C7—C8—C9—C10	0.3 (3)
C1—C2—C3—F	−176.31 (14)	C8—C9—C10—C11	0.1 (3)
C1—C2—C3—C4	−0.1 (2)	C9—C10—C11—C12	−0.2 (3)
F—C3—C4—C5	176.22 (13)	C8—C7—C12—C11	0.5 (2)
C2—C3—C4—C5	0.1 (2)	C4—C7—C12—C11	−178.35 (14)
F—C3—C4—C7	−3.4 (2)	C10—C11—C12—C7	−0.1 (3)
C2—C3—C4—C7	−179.51 (14)	C6—C1—C13—C14	19.40 (19)
C3—C4—C5—F'	177.4 (3)	C2—C1—C13—C14	−161.26 (13)
C7—C4—C5—F'	−3.0 (3)	C6—C1—C13—C15	−102.98 (15)
C3—C4—C5—C6	0.3 (2)	C2—C1—C13—C15	76.36 (16)
C7—C4—C5—C6	179.84 (13)	C1—C13—C15—O	77.34 (16)
C2—C1—C6—C5	0.5 (2)	C14—C13—C15—O	−47.97 (18)
C13—C1—C6—C5	179.81 (13)	C1—C13—C15—N2	−99.18 (14)
F'—C5—C6—C1	−177.7 (3)	C14—C13—C15—N2	135.52 (13)
C4—C5—C6—C1	−0.5 (2)	C17—C16—N1—N2	179.62 (13)
C3—C4—C7—C12	−44.0 (2)	C18—C16—N1—N2	1.1 (2)
C5—C4—C7—C12	136.45 (16)	O—C15—N2—N1	5.0 (2)
C3—C4—C7—C8	137.17 (16)	C13—C15—N2—N1	−178.54 (12)
C5—C4—C7—C8	−42.4 (2)	C16—N1—N2—C15	−169.44 (14)
C12—C7—C8—C9	−0.6 (2)		

### Hydrogen-bond geometry (Å, °)

**Table:**

*D*—H···*A*	*D*—H	H···*A*	*D*···*A*	*D*—H···*A*
N2—H01···O^i^	0.87 (3)	2.24 (3)	3.0633 (17)	158 (2)
N2—H01···N1^i^	0.87 (3)	2.45 (2)	3.0632 (17)	127.7 (19)
C6—H6···F^ii^	0.95	2.45	3.3537 (18)	159
C2—H2···O^i^	0.95	2.52	3.3816 (19)	150
C18—H18A···O^i^	0.98	2.29	3.251 (2)	166

Symmetry codes: (i) *x*+1/2, −*y*+1, *z*; (ii) *x*, *y*−1, *z*.
